# Disseminated Tuberculosis Presenting as Chronic Orchiepididymitis in a Military Trainee: A Case Report and Review of the Literature

**DOI:** 10.1155/2018/7316097

**Published:** 2018-07-29

**Authors:** Michael U. Williams, Ashley Burris, Amy Zingalis, David A. Lindholm, Brian K. White

**Affiliations:** ^1^Department of Medicine, San Antonio Military Medical Center, Joint Base San Antonio-Fort Sam Houston, TX, USA; ^2^Department of Pathology, San Antonio Military Medical Center, Joint Base San Antonio-Fort Sam Houston, TX, USA; ^3^Infectious Disease Service, Wright-Patterson Medical Center, Wright-Patterson Air Force Base, OH, USA; ^4^Infectious Disease Service, San Antonio Military Medical Center, Joint Base San Antonio-Fort Sam Houston, TX, USA

## Abstract

Orchiepididymitis is a clinical diagnosis. The acute form secondary to sexually transmitted or enteric pathogens is well known to primary care providers. However, chronic orchiepididymitis may be secondary to genitourinary tuberculosis (TB), and physicians in countries with a low prevalence of TB might not consider it in their differential diagnosis. Indeed, cognitive errors, such as anchoring or availability bias, may contribute to a delayed diagnosis of genitourinary TB. We present a case of chronic orchiepididymitis as a result of disseminated TB in a Cameroonian male who was visiting the United States for military training. He experienced diagnostic delay and was ultimately diagnosed by orchiectomy. Early consideration of a diagnosis of TB for chronic or recurrent orchiepididymitis in a patient with epidemiologic risk factors is of utmost importance because delayed diagnosis could lead to organ loss.

## 1. Introduction

Despite advances in diagnostics, treatment, and prevention, tuberculosis (TB) remains a global problem, infecting one-third of the world's population [1]. *Mycobacterium tuberculosis* is an aerobic bacillus that typically infects humans via inhalation. TB is capable of surviving in a latent state for many years and can spread hematogenously to distant sites [2]. Although genitourinary TB is not an uncommon manifestation of extrapulmonary TB, physicians in countries of low TB prevalence may not consider it in the differential diagnosis of a young male presenting with chronic or recurrent orchiepididymitis. We report an illustrative case of a Cameroonian male who developed chronic orchiepididymitis as a result of disseminated TB, which was diagnosed by orchiectomy while visiting the United States (US) for military training.

## 2. Case Presentation

A 23-year-old male from Cameroon presented with acute-onset, right-sided scrotal pain and swelling while in the US for military training. He denied current or prior sexual activity, associated penile discharge, hematuria, fevers night sweats, weight loss, or cough. Urinalysis revealed pyuria, follow-on culture was not performed, and urine nucleic acid amplification testing (NAAT) was negative for *Neisseria gonorrhoeae* and *Chlamydia trachomatis*. He was diagnosed with acute epididymitis and treated with ceftriaxone and doxycycline. His symptoms improved, only to recur 6 weeks later, prompting a repeat ultrasound that revealed persistent epididymitis. Repeat urinalysis (no culture) and gonorrhea and chlamydia NAAT were negative. He was given an empiric 30-day course of ciprofloxacin, which improved the pain, but the swelling persisted.

**Figure 1:**
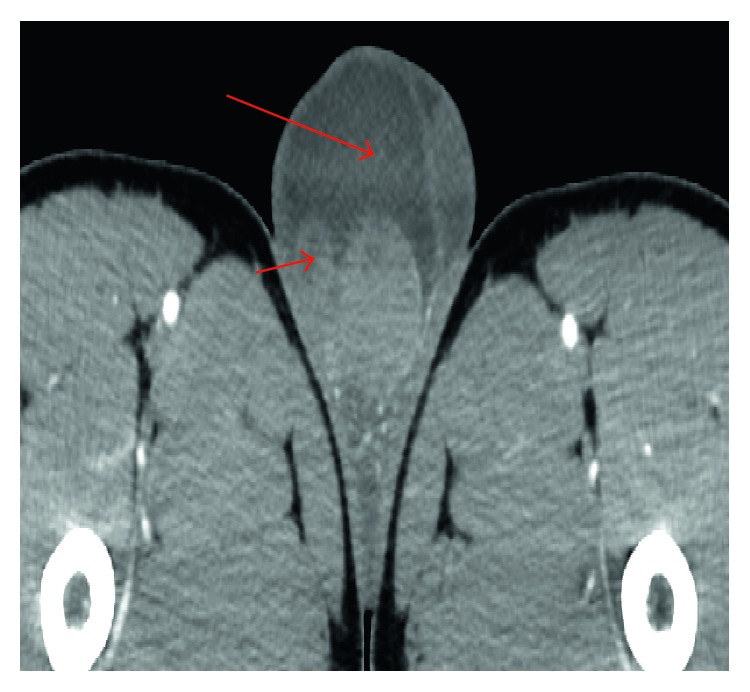
Axial computed tomography (CT) image, depicting a heterogeneous right testicle, with associated right scrotal fluid collection (long arrow) and inflamed right epididymis (short arrow).

Five months after his initial evaluation, he returned with another recurrence of symptoms. Physical exam again revealed prominent right-sided scrotal swelling and tenderness. An ultrasound demonstrated right-sided epididymitis with possible necrosis, increasing complexity of the associated hydrocele, and new concerns for a focal scrotal abscess. This was further evaluated with a contrasted computed tomography (CT) of the chest, abdomen, and pelvis, which revealed a complex right-sided scrotal fluid collection (Figure 1) in addition to an enlarged and heterogeneous prostate, an enlarged juxtaesophageal lymph node, and right lung apical nodular scarring. Although TB was considered in the differential diagnosis, a unilateral orchiectomy was performed, as malignancy was also high on the differential given his age.

**Figure 2:**
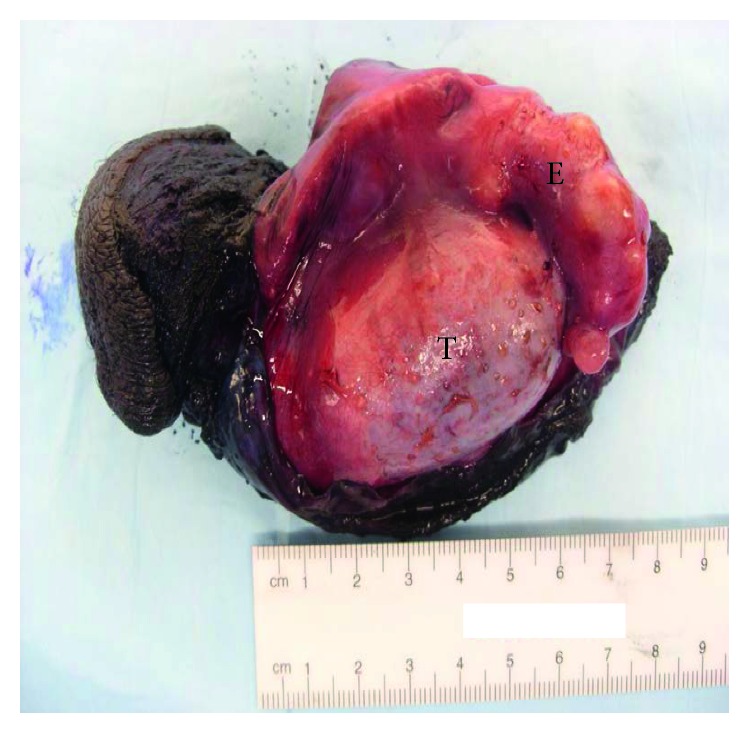
Opened scrotal sac with the exposed testicle (T) and enlarged and nodular epididymis (E).

**Figure 3:**
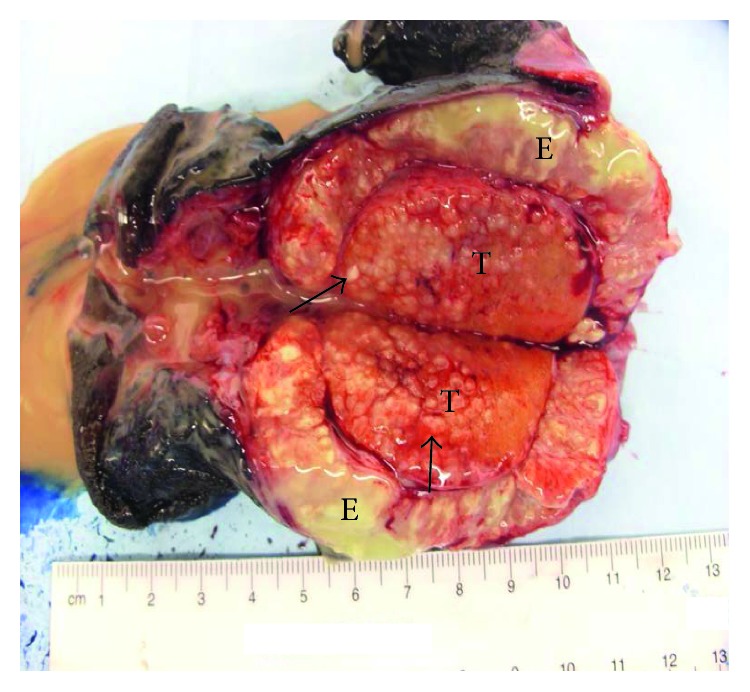
Bisected epididymis (E) and testicle (T), depicting a yellow-tan exudate and miliary deposits (arrows).

**Figure 4:**
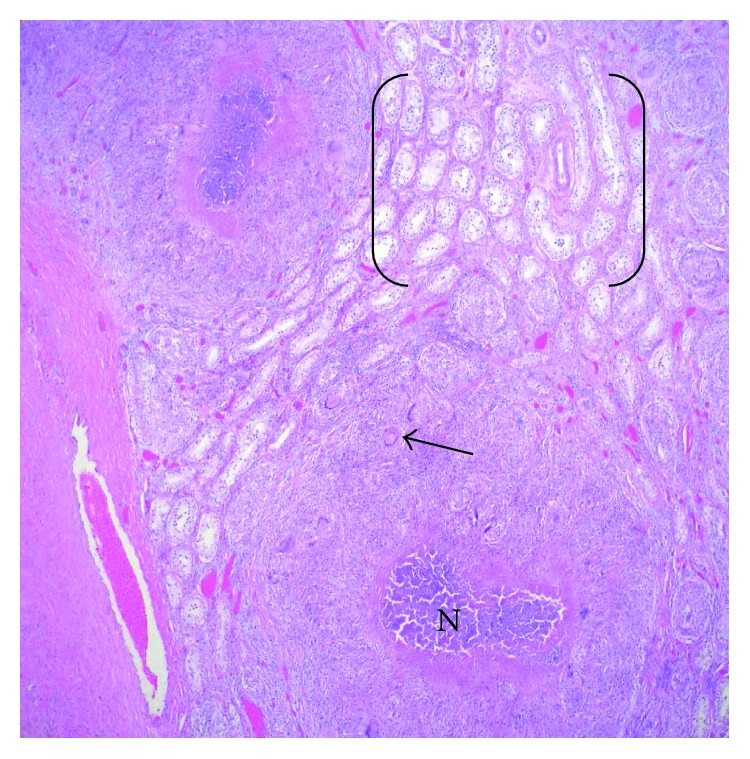
H&E, 4x. Necrotizing granulomatous inflammation in a miliary-type distribution, with multinucleated giant cells (histiocytes, arrow), central necrosis (N), and intervening, uninvolved, normal testicular tubules (brackets).

Grossly, the epididymis was enlarged (6.0 × 1.7 × 1.5 cm; Figure 2), with caseating necrosis and miliary deposits within the testicle and epididymis (Figure 3). There was an accompanying large scrotal abscess. The histologic sections showed diffuse necrotizing granulomas, with giant cell formation in the scrotum, testis, and epididymis (Figure 4). The testicular parenchyma stained positive for acid-fast bacilli (AFB), and a surgical-scrotal aspirate NAAT using the GeneXpert MTB/RIF assay (Cepheid, Sunnyvale, CA) was positive for *Mycobacterium tuberculosis* (no rifampin resistance was detected). Urine AFB stain and urine NAAT were negative for TB; ultimately, the urine AFB culture was positive for TB. Despite a normal chest X-ray and reported lack of pulmonary symptoms, his sputum was also positive for TB by stain, NAAT, and culture. Antimicrobial susceptibility testing revealed no resistance to first-line agents. An immunoassay for human immunodeficiency virus (HIV) was nonreactive.

He was started on rifampin, isoniazid, pyrazinamide, and ethambutol. However, due to the development of hepatotoxicity 12 days into therapy without evidence of viral hepatitis, he was transitioned to ethambutol, levofloxacin, and intravenous amikacin. On treatment day 15, his sputum smears converted to negative. After resolution of his drug-induced liver injury on treatment day 26, amikacin was discontinued in favor of rifampin, and he was recalled to Cameroon soon thereafter on an all-oral, three-drug regimen.

## 3. Discussion

Extrapulmonary TB accounted for 18.7% of TB cases in the US from 1993 to 2006; of those extrapulmonary cases, 6.5% involved the genitourinary tract [3], although genitourinary involvement has been reported to be present in as high as 42.9% of cases in other countries [4]. The diagnosis of genitourinary TB is challenging and requires a high level of clinical suspicion based on the epidemiologic context. Given the low prevalence of TB in the US and the fact that patients with extrapulmonary TB often lack the classic symptoms of pulmonary TB, US physicians may have a low clinical suspicion for genitourinary TB [5]. Unfortunately, the late diagnosis of TB orchiepididymitis can result in organ loss [6].

Risk factors for genitourinary TB include foreign birth, HIV infection, hemodialysis, and kidney transplantation [7–9]. In 2016, over 9,000 TB cases were reported in the US, and 68% of the cases occurred among foreign-born persons, making this a key trigger to raise one's clinical suspicion [10]. Additionally, suspicion for TB should be higher in patients whose symptoms fail to resolve or who have recurrent symptoms after empiric treatment for common causes of acute epididymitis [8].

The diagnostic delay in our patient was primarily the result of failing to consider TB in the differential diagnosis. In clinical practice, cognitive error is more common than knowledge gap error and commonly leads to misdiagnosis [11]. Cognitive errors (e.g., anchoring, availability bias, representativeness bias, and premature closure) likely contributed to his delayed diagnosis. Chronic orchiepididymitis is variably defined as at least 6 weeks to 3 months of discomfort and/or pain localized to the scrotum [12, 13]. In a patient presenting with such a clinical syndrome, it is important to maintain a broad differential diagnosis, including both infectious and noninfectious causes: TB, bacillus Calmette–Guérin, filariasis, leishmaniasis, brucellosis, syphilis, chlamydia, paracoccidioidomycosis, malignancy, autoimmune diseases, obstruction, medication adverse effects, trauma, postinfectious, and idiopathic [12–15]. Noninvasive tests directed by epidemiologic clues, such as three or more early-morning urine AFB cultures [8] in a foreign-born male, may allow for early diagnosis and successful pharmacotherapy, eliminating the need for orchiectomy. However, testicular malignancies can present similarly, are most common among males 15 to 34 years of age [16], and may be cured by surgical excision [17–19]. If a noninvasive approach fails to diagnose genitourinary TB, surgery may be necessary in order to confirm the diagnosis and rule out malignancy [6].

Although the GeneXpert assay is approved by the Food and Drug Administration for pulmonary specimens only, its use as a tool in the diagnosis of extrapulmonary TB has been studied. Meta-analyses show a wide range of sensitivity (0–100%, median 22–83%) and a narrower range of specificity (73–100%, median 98–100%) [20–22]. The variability in sensitivity is secondary to differences in study design, patient population, gold standard, sample processing, anatomic source of the sample, tissue or fluid type of the sample, sample volume, nonhomogeneous distribution of mycobacteria within the sample, mycobacterial burden, and AFB smear status [9,20–22]. Although it should not be used to rule out extrapulmonary TB given its poor sensitivity, the off-label use of the Xpert assay can be very helpful in the rapid detection of extrapulmonary TB given its high specificity and its applicability to samples obtained via noninvasive (e.g., voided) or minimally invasive techniques (e.g., image-guided aspiration) [21, 22]. Early diagnosis can facilitate early therapy and—in some cases—may even prevent premature surgery. In our patient, the urine NAAT was negative, but the NAAT from the surgical scrotal aspirate was positive and triggered the initiation of anti-TB therapy.

Our case highlights the importance of maintaining a broad differential diagnosis to avoid cognitive errors and using the patient's epidemiologic context to increase clinical suspicion for genitourinary TB. Although not pursued in our case early enough to avoid surgery, early consideration of microbiologic or molecular testing of nonsurgical specimens may facilitate diagnosis and optimize outcomes.
